# Chemical analysis of dentin surfaces after Carisolv treatment

**DOI:** 10.4103/0972-0707.57636

**Published:** 2009

**Authors:** Veena S Pai, Roopa R Nadig, TG Jagadeesh, G Usha, J Karthik, KS Sridhara

**Affiliations:** Departments of Conservative Dentistry and Endodontics, Dayananda Sagar College of Dental Sciences, Bangalore, India

**Keywords:** Carisolv 2, Raman spectroscopy, scanning electron microscopy

## Abstract

**Aims and Objectives:**

This study was done to characterize the surface chemistry after caries excavation with burs and Carisolv 2, by analyzing the relative amounts of organic and inorganic content, and also to analyze the penetration of the adhesive after etching and bonding using Micro Raman spectroscopy.

**Materials and Methods:**

Twenty extracted molars with caries were distributed into the following groups and treated accordingly. Group 1-excavation with bur (10 teeth), and Group 2-excavation using Carisolv 2 (10 teeth).

**Results and Conclusion:**

Spectroscopic analysis showed that there was no significant difference in the chemical composition of the tooth between the groups after excavation (*P* > 0.05) either with bur or with Carisolv. The penetration of the dentin bonding resin in all samples of the Carisolv group was up to 15μm, whereas, in the bur group it was upto 10μm in few samples. Scanning Electron Microscopic analysis showed the surfaces of the Carisolv-treated dentin to be free of the smear layer, with open tubules, whereas, the dentin surfaces of the bur group showed surfaces covered with a smear layer. In the Carislov group the resin tags were found comparatively deeper than in the bur excavation group. In both the groups the integrity of the remaining dentin surfaces were maintained chemically and morphologically.

## INTRODUCTION

Since the invention and application of rotary instruments, the operative treatment of carious lesions has often resulted in considerable removal of tooth structure.[1] With advances in technology and material science there is a paradigm shift from the traditional surgical model (drill and fill) to the modern medical model of care, involving caries risk assessment, alteration of cariogenic environment, potential tissue remineralization, and minimal tooth preparation.

Dentinal caries has an outer layer contaminated by bacteria causing a non-remineralizable necrotic collagen matrix, and an inner layer, where the bacteria are less frequently seen; the collagen is reversibly denatured, but retains the cross banded ultrastructure and if the acid attack is removed it has the potential to remineralize. Preserving this layer of dentin would be a conservative approach without exposing the pulp, preventing overzealous preparations. To achieve this, various newer caries excavation techniques have been suggested like air abrasion, air polishing, ultrasonic, sono abrasion, lasers, enzymes, and the chemomechanical caries removal technique. Banerjee *et al*., in a review, concluded that except for the rotary burs and chemomechnical systems, none of the techniques were effective in removal of dentinal caries. [1]

Chemomechanical caries removal technique is a noninvasive hand excavation method with the aid of a chemical gel. Carisolv 2 is a chemomechanical agent, marketed in two solutions, which are mixed prior to application. Solution 1 contains three amino acids (leucine, lysine, glutamic acid) and NaOH, NaCl in purified water, Solution 2 contains 0.5% NaOCl.

The mode of action of chemo mechanical caries removal systems (CMCRs) is by chlorination of the partially degraded collagen and cleavage by oxidation of glysine residue, which results in collagen fibril disruption making the collagen fibrils more friable and easy to remove. The hard unaffected dentin is not removed. The advantage of chemomechanical agents is that it selectively removes softened dentin and conserves the tooth structure, thereby lessening the chance of iatrogenic pulp exposure. It is claimed to be a painless procedure, which is bio compatible, with no pulp reaction. It also aids in bonding adhesive restorations.[2]

The outcome of bonding between tooth and restorative materials, which depends on the surface property of prepared / treated dentin, needs a greater understanding of the surface alteration of dentin after Carisolv treatment and its consequent effect on hybrid layer formation. Hence, this study was done to characterize the surface chemistry after caries excavation with burs and Carisolv, by analyzing the relative amounts of organic and inorganic content, and also to analyze the penetration of the adhesive after etching and bonding, using Micro Raman spectroscopy.

## MATERIALS AND METHODS

Twenty extracted molars with caries were used in this study and were distributed into the following groups and treated accordingly.

### Group distribution

Group 1 - Excavation with bur (10 teeth)

Group 2 - Excavation using Carisolv 2 (10 teeth)

#### Group 1 - Excavation with bur

Caries were mechanically removed with a low-speed round carbide bur under a water coolant until all the soft caries were removed. The prepared cavity surfaces were studied under Micro Raman spectroscopy. The prepared surfaces were then bonded with self-etch two-step adhesive (Unifil Bond) and sectioned with a diamond disk and water coolant.

#### Group 2- Excavation with Carisolv

The two components were mixed according to the manufacturer's instruction and the required amount was taken in a dappen dish. With the multi-star instrument the gel was applied to the carious dentin to soak it generously. A waiting period of 30 seconds, as recommended by the manufacturer, for the chemical process to soften the caries, was followed. The process was repeated until the solution remained clear without turning cloudy and the softened dentin was excavated with the multi star instrument. After completion of excavation, the surface was wiped with a moist cotton pellet.

Specimen surfaces of both the groups were analyzed under Micro Raman Spectroscopy for the organic and inorganic content, after excavation with bur and Carisolv. All the specimens were bonded using Unifil Bond (two-step self-etch adhesive) according to the manufacturers instructions, and observed under Micro Raman Spectroscopy to look for any change in the dentin integrity and penetration of the adhesive into the dentin.

### Scanning electron microscope observation

Fifty percent of the samples from both the groups were observed under a scanning electron microscope (SEM), to analyze the morphological changes and the dentin surface after excavation. After application of the Unifil bond, a similar number of specimens were observed under SEM for penetration of resin tags into the dentin.

### Instrumentation for micro Raman spectroscopy

Micro Raman spectra were recorded with a Jasco Raman Spectrometer equipped with Olympus Lenses.

The excitation source was a He-Ne laser, operating at 632.8 nm. After passing through the band pass filter and condensing optics, the 3 MW power laser was incident upon the samples. The samples were placed on a glass slide and focused under a ×100 microscope objective lens and spectra were acquired at the surface of the hybrid layer, 10 μm and 20 μm into the dentin. Spectra were obtained over the spectral regions from 600 cm^–1^ to 1800 cm^–1^ with an integration time of 120 seconds.

#### Quantifying the spectral readings

Spectral data from the data interfaces were compared with the reference spectra of pure adhesive and normal dentin.

### Evaluation of demineralization and degradation of dentin

**Graph 1:**
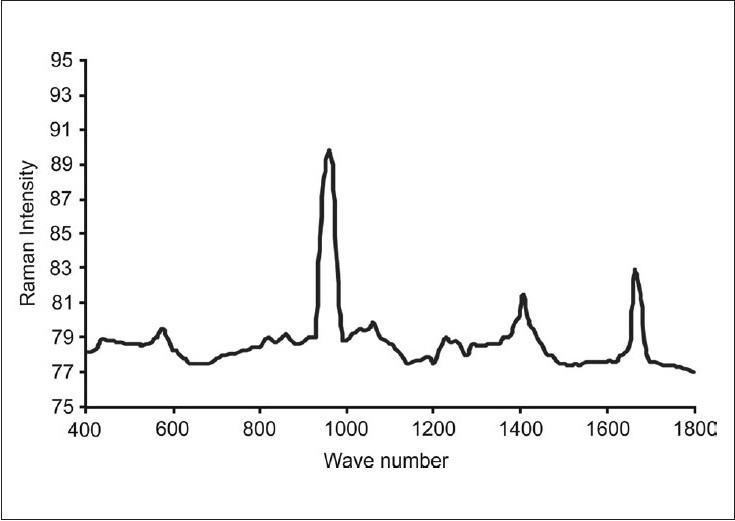
MRS spectra obtained for normal dentin - peaks at 960 cm^−1^ (phosphate) and 1666 cm^−1^ (amide I) used as internal standards

The intensity of peak 960 cm^–1^, representative of the mineral content of normal dentin by PO_4_ vibration and 1666 cm^–1^ representative of organic content of dentin by amide I, were taken as standards [Graph 1], to compare the readings of the same peaks after bonding, to analyze the changes in the organic and inorganic components in the dentin samples.

### Statistical analysis

Students ‘T’ test was performed to analyze the difference in chemical structure between the groups.

## RESULTS

There was no significant difference between the two groups in their chemical composition after excavation (*P* > 0.05). The penetration of the dentin bonding resin in all samples of the Carisolv group was up to 15 μm, whereas, in the bur group it was up to 10 μm in few samples. One of the samples in Carisolv treated group showed presence of Carisolv and caries. Scanning electron microscopic analysis showed the surfaces of the Carisolv-treated dentin, free of the smear layer, with open tubules, whereas, the dentin surfaces of the bur group showed the surfaces covered by the smear layer. In the Carislov group the resin tags were found comparatively deeper than in the bur excavation group. In both the groups the chemical and morphological integrity of the remaining dentin surfaces were maintained.

## DISCUSSION

Although the action of chloramines was explored in an early system for chemical caries removal (Caridex, USA), this system had many limitations, such as, difficult clinical handling, large volume of solution needed, short shelf-life of opened packages, and time required to complete caries removal.[3]

An improved version of the chemomechanical system was Carisolv 1, introduced in 1997, by team Mediteam dental AB (Sweden),[1,4] which has now been improved and marketed as Carisolv 2,[5,6] consisting of two syringes, which are mixed before usage. Syringe 1 contains three amino acids (leucine, lysine, glutamic acid), NaOH, NaCl in purified water. Syringe 2 contains 0.5% NaOCl.

When the two Carisolv components (two syringes) are mixed prior to treatment, at pH 11, stable monochlorinated forms of these amino acids are formed. The chlorine atom of the hypochlorite is transferred to the amino group of each amino acid, and in this way it is made less reactive and less aggressive to healthy tissue. The alkaline pH ensures suppression of formation of more reactive chlorine species, such as, dichlorinated amines and hypochlorous acid. Besides, in a reducing environment of an alkaline solution, chlorination rather than oxidation of an organic molecule is favored (the oxidative property of the hypochlorite is suppressed). By including the three chloroaminoacids with different side chain properties - positively charged, negatively charged, and hydrophobic - it is ensured that they will electrostatically attract all three possible protein patches, not only collagen, but also all proteins and large organic molecules. It is still not certain how the disruptive power of chlorine is exerted in the target tissue, but it probably occurs at non-covalent bonds, like hydrophobic or van der Waals interactions. These, although they are individually weak, are present in large numbers. The specificity toward proteins introduced by amino acid chlorination gives a protection potential for the healthy dentin, which is largely non-proteinaceous and has as its major constituent the mineral, hydroxyapatite. Also, the high pH stabilizes the mineral structure by decreasing its solubility (this is favored at low pH).[3]

Numerous chemical studies have been done in an effort to understand the dentine surfaces after various treatments using FT-Raman Spectroscopy, Fourier transform infra red spectroscopy, and Laser ablation inductively coupled plasma mass spectroscopy. Micro Raman spectroscopy was used in this study to analyze the chemical composition of dentine as it is considered to be useful, having a cost effective analytical technique with high specificity. Here the acquired spectra are attributed to molecules rather than to single elements and the laser beam can be focused to a very small spot size. A high spatial resolution at the sample surface can be achieved without dehydration of the sample and measurements can be taken at room temperature.

In this study for Raman spectra of normal dentin the band at 960 cm^− 1^ - representative of mineral content by phosphate vibration, and 1657cm^– 1^ - organic component band caused by the amide groups, as found through various studies analyzing chemical content of enamel and dentin, are used as internal standards. Raman spectra reflect chemical composition with only bands that change their polarity during vibration, which are detected.[7]

**Graph 2:**
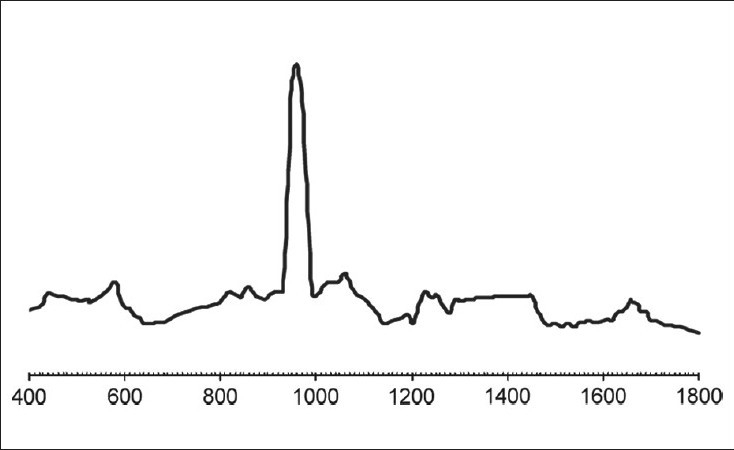
MRS spectra obtained from dentin surfaces after excavation by bur. Peaks are similar to normal dentin

**Graph 3:**
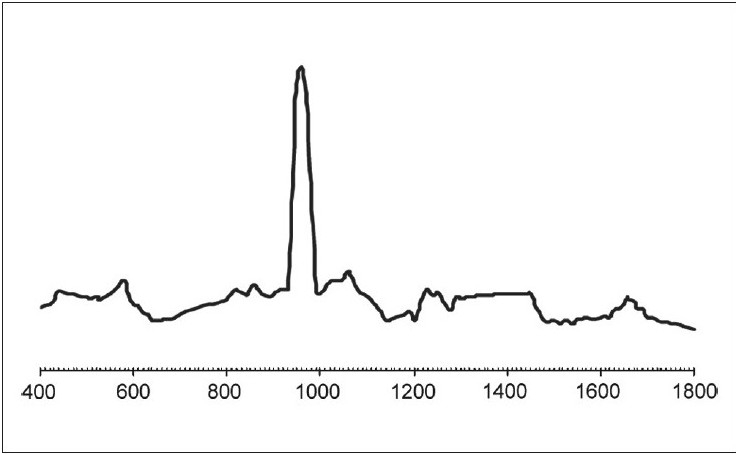
MRS spectra obtained from dentin surfaces after excavation using Carisolv. Peaks are similar to normal dentin

**Graph 4:**
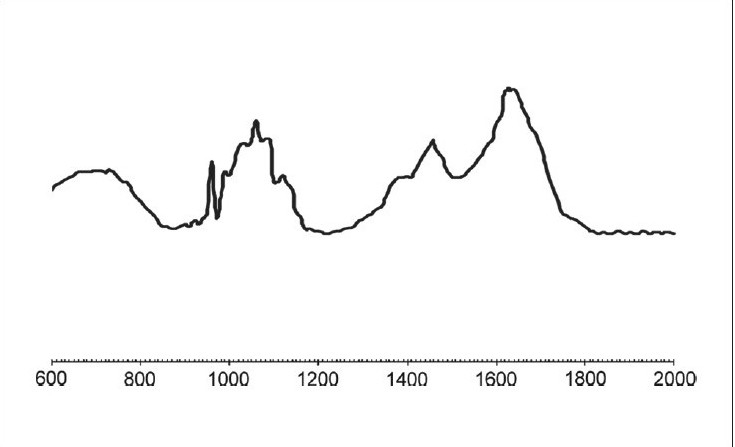
MRS spectra obtained from dentin surfaces after excavation by Carisolv in which caries and Carisolv both remain

The results of our study showed no significant difference between both the groups in their organic or inorganic contents after caries excavation, which is in agreement with the previous study by Arvidsson on chemical and topographical analysis, which showed no major differences between cavities excavated with burs or Carisolv[8][Graphs 2 and 3]. In one sample both caries and Carisolv were remaining, which could be attributed to improper excavation after Carisolv application [Graph 4].

**Graph 5:**
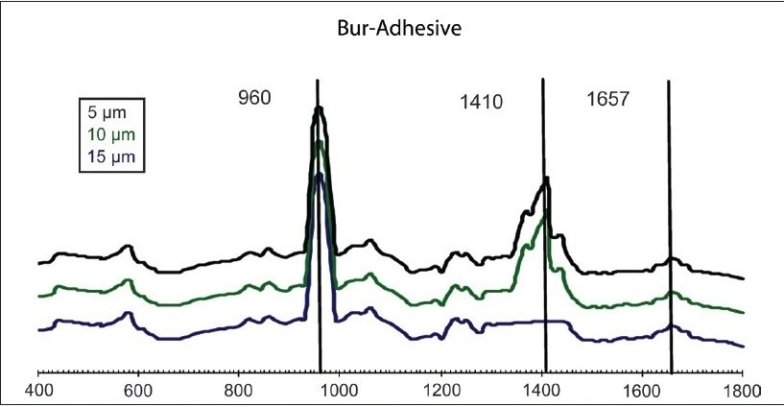
MRS spectra obtained from dentin surfaces after excavation by bur and after adhesive application

**Graph 6:**
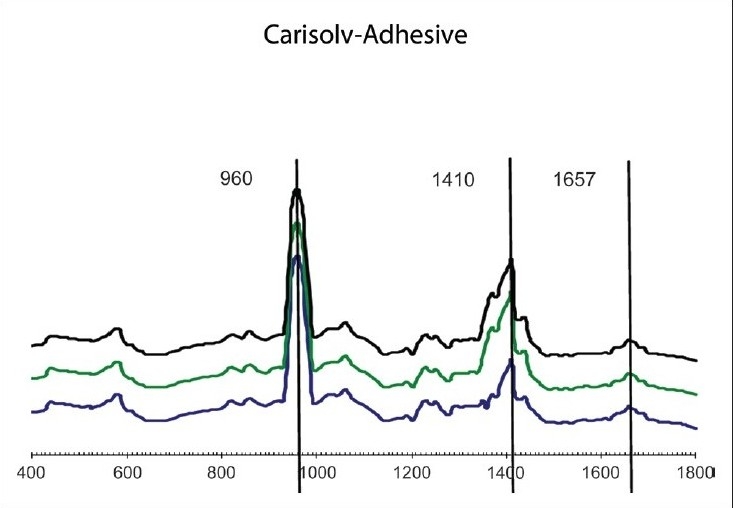
MRS spectra obtained from dentin surfaces after excavation using Carisolv, and after adhesive application

**Figure 1:**
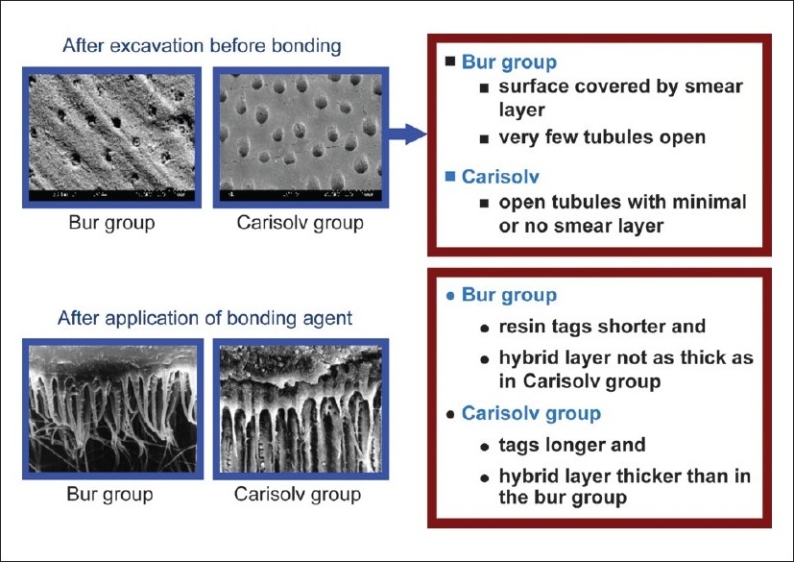
After excavation before bonding; after application of bonding agent

Second part of the study involved analyzing the depth of penetration of the bonding agent after application of Unifil bond-2 step SEA. The results of our study showed significant difference between the groups, with the bur group resin tags (10 μm, [Graph 5]) showing lesser penetration when compared to the Carisolv group (15 μm, [Graph 6]). The SEM observation supported these results [Figure 1]. The increased depth of penetration of the adhesive into the dentin was possibly because of the more effective smear layer removal by Carisolv. The NaOCl in Carisolv helps in the removal of the smear layer, thereby leaving a rough surface with open tubules and increased interfibrillar spaces, hence increasing the depth of penetration and a thicker hybrid layer.[9] However, that the increase in the depth of penetration and a thicker hybrid layer has any bearing on the bond strength is controversial. There are several studies that say 2-5 μm tags are adequate for good bonding. Whether increased penetration observed in this study would influence the longevity of the bond has to be ascertained.

## CONCLUSION

Under the limitations of this study, no significant difference in the chemical structure of dentin surfaces was observed between bur excavated and Carisolv excavated dentin. Depth of penetration of bonding resin was significantly more in the Carisolv group compared to the bur group. The relationship between the depths of penetration of the resin to the longevity of the bond in the Carisolv-treated teeth needs to be ascertained by further *in vitro* and*in vivo* studies.
